# Analysis of mir-9 Expression Pattern in Rat Retina during Postnatal Development

**DOI:** 10.3390/ijms22052577

**Published:** 2021-03-04

**Authors:** Etelka Pöstyéni, Andrea Kovács-Valasek, Péter Urbán, Lilla Czuni, György Sétáló, Csaba Fekete, Robert Gabriel

**Affiliations:** 1 Department of Experimental Zoology and Neurobiology, University of Pécs, 7624 Pécs, Hungary; etelka91@gamma.ttk.pte.hu; 2 János Szentágothai Research Centre, 7624 Pécs, Hungary; urpe.89@gmail.com (P.U.); czuni.lilla@gmail.com (L.C.); fekete@gamma.ttk.pte.hu (C.F.); 3 Department of Medical Biology, Medical School, University of Pécs, 7624 Pécs, Hungary; gyorgy.setalo.jr@aok.pte.hu

**Keywords:** mir-9, retina, postnatal development, in situ hybridization, qPCR, next-generation sequencing, synaptotagmin-17, OneCut2

## Abstract

It is well established that miR-9 contributes to retinal neurogenesis. However, little is known about its presence and effects in the postnatal period. To expand our knowledge, miRNA-small RNA sequencing and in situ hybridization supported by RT-qPCR measurement were carried out. Mir-9 expression showed two peaks in the first three postnatal weeks in Wistar rats. The first peak was detected at postnatal Day 3 (P3) and the second at P10, then the expression gradually decreased until P21. Furthermore, we performed in silico prediction and established that miR-9 targets OneCut2 or synaptotagmin-17. Another two microRNAs (mir-135, mir-218) were found from databases which also target these proteins. They showed a similar tendency to mir-9; their lowest expression was at P7 and afterwards, they showed increase. We revealed that miR-9 is localized mainly in the inner retina. Labeling was observed in ganglion and amacrine cells. Additionally, horizontal cells were also marked. By dual miRNA-in situ hybridization/immunocytochemistry and qPCR, we revealed alterations in their temporal and spatial expression. Our results shed light on the significance of mir-9 regulation during the first three postnatal weeks in rat retina and suggest that miRNA could act on their targets in a stage-specific manner.

## 1. Introduction

MiRNAs are members of small endogenous noncoding RNA molecules with a prominent regulatory function on gene expression and show tissue-specific action in vertebrates [1]. Many studies in the last decade have been devoted to revealing the presence and regulatory role of microRNAs in many diversified processes of nervous system development. It is well established that they can contribute to differentiation, proliferation and apoptosis, and their importance in various physiological and pathological conditions is also being scrutinized [2,3,4,5,6,7]. Emerging data suggest that parallel to the chronological order of retinal cell types differentiation, miRNAs are also expressed differentially during development and have a prominent negative regulatory function via mRNA degradation or repression of transcription [2,6,7,8,9,10,11,12,13,14].

In the development process of the central nervous system (CNS), sequential actions contribute to the differentiation of neuron and glial cells [15,16,17]. The mammalian retina is a characteristic multi-layered tissue and, as part of the CNS, has a fundamental role in transferring the visual signals to the brain. Its well-organized structure consists of three neural and two synaptic layers that are formed during retinal neurogenesis. This developmental process is extremely sophisticated and precisely scheduled. Sequential actions instruct multipotent retinal progenitor cells (RPC) to generate the seven principal cell types via numerous regulatory factors, then these differentiate into subtypes and connect to each other [8,18,19,20,21,22]. The retinal cells can be divided into two groups: early-born cells (amacrine cells, retinal ganglion cells, cones, horizontal cells), which mostly appear during embryonic development, and late-born cells (bipolar cells, rods, photoreceptor cells, Müller cells), mostly generated postnatally [19,20,23,24]. In recent years, considerable attention has been paid to the highly conserved mir-9, which was expressed specifically in the neurogenic regions of the brain, besides being present in the peripheral nervous system [4,13,25,26,27,28]. Loscher and his colleagues made an analysis where they compared the miRNA expression patterns of the brain and the retina. They found that 47 miRNAs were twofold more enriched in the retina than in the brain, and one of them was mir-9 [3]. Another microarray analysis has shown that from 78 miRNAs found in adult rat retinas, 21 were retina-specific. Of these 21 miRNAs, 17 had a peak at postnatal Day 10 (P10), and mir-9 was found among them [29]. The contribution of mir-9 in neurogenesis and in angiogenesis has been described in the developing brain. The inhibition of mir-9 has been found to result in increased expression levels of TLX and ONECUT transcriptional factors, changes in which lead to VEGF-A overexpression. Novel mir-9 expression in Müller cells of the retinal neuronal stem cells has also been described [28].

Several transcription factors such as members of the T-box transcription factor (Tbx), the Iroquois homeobox (Irx) and the OneCut family were suggested and confirmed to be involved in this process [13]. Mir-9 contributes to many developmental processes such as neurogenesis, neuronal cell proliferation, migration and RPC differentiation [25,26]. Its role in retinal progenitor cell differentiation and proliferation has also been described. Pre-mir-9-treated RPCs showed increased mir-9 expression levels and TLX expression decreased; on the other hand, anti-mir-9 treatment caused increased levels of TLX in RPCs. The downregulation of TLX levels and the overexpression of mir-9 cause enhanced differentiation and decreased RPC proliferation [30]. Other in vitro RPC investigations demonstrated that mir-9 targeted the upstream components of the Janus kinases- signal transducer and activator of transcription protein signaling (Jak–Stat) pathway, namely the transcription factor PTBP1, thus attaining the inhibition of Stat phosphorylation and suppression of astrogliogenesis [31,32]. La Torre et al., described that mir-9 is a key regulator factor which takes part in the competence change of retinal progenitors during development, and shows increasing expression levels between the embryonic and postnatal periods. They found that the expression of three microRNAs (mir-125, let-7, mir-9) increased in progenitors during development, while through overexpression in normal retinas, these microRNAs cause acceleration in development. They have also been described in Dicer knockout (KO) animals, where the overexpression of these miRNAs allows their progression to late progenitors and show higher OneCut2 gene expression. The knockdown of these abovementioned microRNAs results in the same phenotype as Dicer KO animals [13]. MicroRNA array results from ciliary epithelial stem cells in developing and adult retinas have shown that mir-9 is highly expressed in P4 retinas [9]. By molecular analysis, mir-9 was identified as a Müller glia-specific miRNA in the postnatal retina and detected in the middle part of the inner nuclear layer (INL), with an increasing level from P11 to adulthood [33,34]. On the other hand, mir-9 overexpression inhibits glial differentiation at P14 in mice retinas [35]. According to the miRTarBase platform, mir-9 could also target a member of the widely studied synaptotagmin family, namely synaptotagmin-17 (also known as brain/kidney (B/K) protein). The presence of synaptotagmin-17 in the central nervous system, especially in the brain, has been widely confirmed [36,37,38,39,40,41]. These previous investigations have revealed that it is an unusual isoform because of the lack of transmembrane domains, so it is not involved in exocytosis, but instead has a crucial role in intracellular membrane trafficking [37,41,42]. However, little research has been conducted to reveal the developmental distribution and expression alterations of synaptotagmin-17 in the rat retina [43,44]. Although prior studies have thoroughly investigated the role of mir-9 in embryonic retinal neurogenesis, little research has been conducted to reveal its quantity and distribution during postnatal development. The present study was designed to gain deeper insight into molecular regulation networks during postnatal retinal development by characterizing mir-9′s spatial and temporal expression with its potential two targets. We used the user-friendly and widely annotated miRNA-target interaction platforms miRTarBase and TargetScan to reveal the experimentally validated targets of mir-9. For further analysis, OneCut2, a transcriptional factor and synaptotagmin-17, known as a synaptic physiology-related regulator, was selected. For better understanding the expression of these target proteins, we also searched for miRNAs (mir-135, mir-218) which had an effect on their expression in databases and from the literature. Spatiotemporal localization of miRNAs is essential for identification of their targets and helps to explain how these molecules modulate retina function and development. Although the role of mir-9 in cell differentiation has already been described in the retina, the exact change in the quantity and distribution of expression is still unknown during postnatal development.

## 2. Results

**Figure 1:**
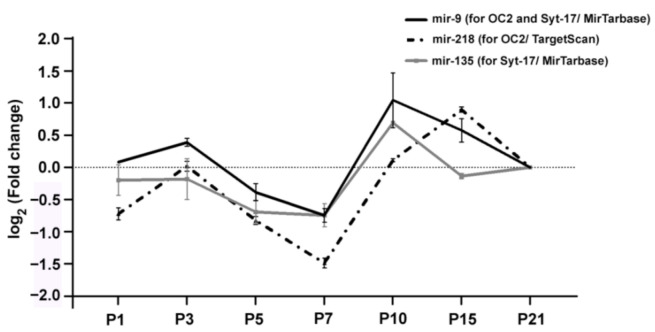
The sequencing results of mir-9 (black line), mir-218 (dashed line) and mir-135 (gray line). Fold changes are represented as log2 fold change compared with P21. OC2, OneCut2; Syt-17, synaptotagmin-17.

We performed a miRNA-Seq study to get deeper insight into the gene expression regulation at the miRNA level of retinal late-born cell development (GSE159168). From this study, we took postnatal expression results for mir-9 and two other OneCut2- and synaptotagmin-17-related miRNAs (mir-135-synaptotagmin-17, mir-218-OneCut2) (Figure 1).

**Figure 2:**
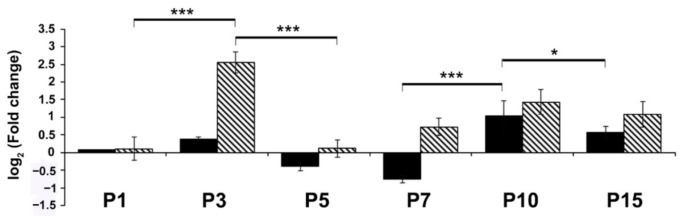
The altered expression of miR-9 revealed by RNA-sequencing (black) and RT-qPCR (striped). The change in miR-9 expression was calculated with U6snRNA as a reference gene for normalization. Fold changes are represented as log2 fold change compared with P21 (* *p* < 0.05, *** *p* < 0.001; Table S1).

Their expressions had almost the same tendency: the lowest values occurred at P7 and the peak at P10. Mir-9 and mir-218 had a smaller peak at P3; on the other hand, mir-135 had only one peak at P10, where mir-9 also showed the highest expression level. qPCR measurements mostly supported the sequencing data concerning mir-9 (Figure 2; Table S1).

**Figure 3:**
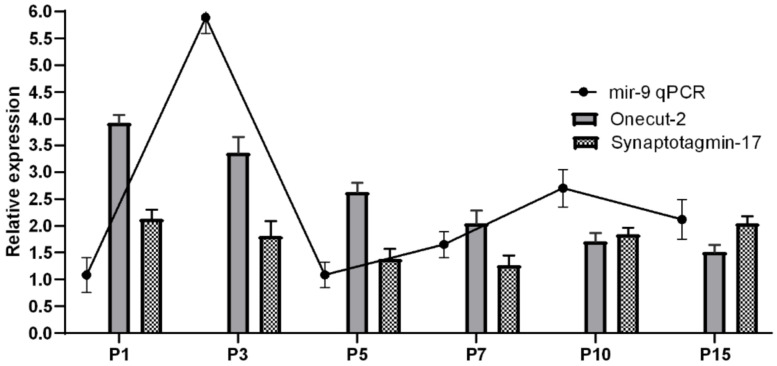
Relative expression levels of miR-9 target genes: OneCut2 (gray), synaptotagmin-17 (spotted) and mir-9 (black line) determined by RT-qPCR. Target genes expression levels were normalized against glyceraldehyde-3-phosphate dehydrogenase (GAPDH) as an internal control gene. The change in mir-9 expression was calculated with U6snRNA as a reference gene for normalization. Expression was compared with P21.

We observed that mir-9 peaked two times in the first three postnatal weeks: a more than twofold expression was measured at P3 and at P10 (*p* < 0.0001; *p* = 0.0087). The patterns of mir-9 and its targets predicted by their quantitative expression changes were also revealed by qRT-PCR (Figure 2 and Figure 3; Table S1).

miRTarBase target detection software indicated that mir-9 targets OneCut2 based on a reporter assay study [45]. At the same time, targeting of synaptotagmin-17 was supported by qPCR examination [46]. To reveal the expression patterns of these potential targets of mir-9, RT-qPCR was performed. The mRNA expression of OneCut2 showed a gradually decreasing tendency during the examined period (Figure 3, gray); in the first period (P1–P7), this trend was more intensive (*p* < 0.01) than after P10.

**Figure 4:**
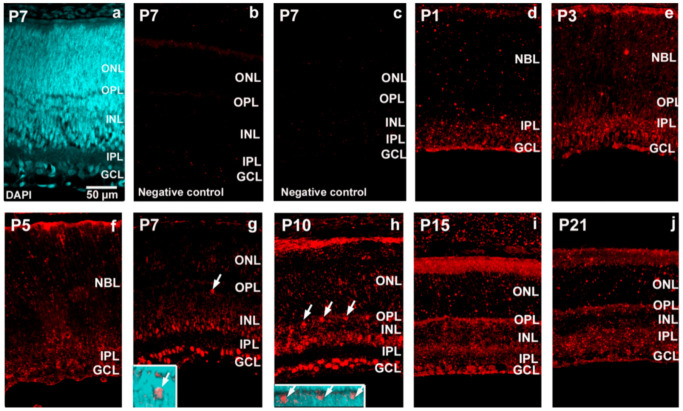
The cellular localization of miR-9 in the Wistar rat retina in the first 3 postnatal weeks by in situ hybridization ((**d**) P1; (**e**) P3; (**f**) P5; (**g**) P7; (**h**) P10; (**i**) P15; (**j**) P21). The retina has been counterstained with 4′,6-diamidino-2-phenylindole (DAPI) (**a**) and different controls (**b**) without anti-mir-9, or (**c**) without anti-digoxigenin (DIG)-horseradish peroxidase are presented. Insert images demonstrate the overlap of mir-9 in situ hybridization (ISH) with DAPI. Arrows indicate horizontal cells at P7 and P10. The scale bar represents 50 µm. GCL, ganglion cell layer; IPL, inner plexiform layer; NBL, neuroblast layer; INL, inner nuclear layer; OPL, outer plexiform layer; ONL, outer nuclear layer.

Additionally, the mRNA level of synaptotagmin-17 was lower, and its changes were less dramatical than those of OneCut2 (Figure 3, spotted). Synaptotagmin-17 had a declining trend from P1 to P7, then rose at P10 and P15; finally, it showed a sharp reduction (Tables S2 and S3). In order to reveal mir-9′s appearance in postnatal Wistar rat retina, a time-course study was carried out in the first 3 weeks after birth, applying in situ hybridization (Figure 4).

**Figure 5:**
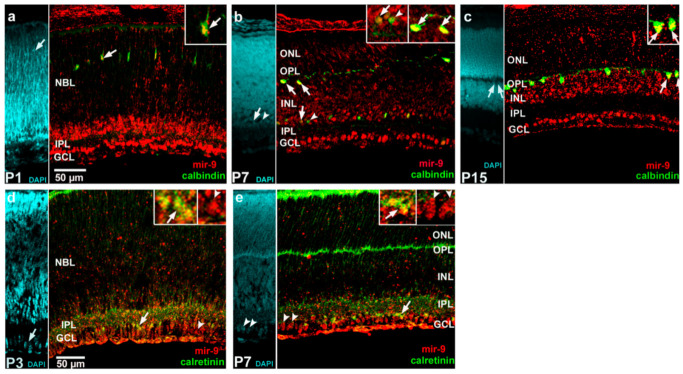
Representative images of miR-9 (red: tyramide signal amplification (TSA) Cy 3) co-detection with neuron-specific marker protein (calbindin D28k or calretinin; green: Alexa Fluor-488) at the indicated postnatal days (mir-9 with calbindin: (**a**) P1; (**b**) P7; (**c**) P15; mir-9 with calretinin: (**d**) P3; (**e**) P7). Insert images demonstrate the overlap or nonoverlap of ISH–dual IHC. Co-localizations are shown by arrows, while non-co-localized cells are demonstrated by arrowheads. The scale bar represents 50 µm. GCL, ganglion cell layer; IPL, inner plexiform layer; NBL, neuroblast layer; INL, inner nuclear layer; OPL, outer plexiform layer; ONL, outer nuclear layer.

The retinal tissue was counterstained with 4′,6-diamidino-2-phenylindole (DAPI) (Figure 4a) and to different controls (Figure 4b, without anti-mir-9; Figure 4c, without anti- digoxigenin (DIG) horseradish peroxidase). The presence of mir-9 was already strong at P1, mainly in the inner part of the retina. The ganglion and amacrine cells were already arranged in layers and showed remarkable hybridization-related tyramide signal amplification (TSA) fluorescence, while faintly stained cells were detected in the inner part of the neuroblast layer (NBL) (Figure 4d). At P3, the TSA signals of supposed ganglion cells and amacrine cells showed slightly increased intensity. Compared with P1 the inner part of the NBL showed lighter labeling with diverse and moderately labeled cells (Figure 4e). By P5, a decline in the staining intensity was detected compared with the earlier stages (Figure 4f). This decreased hybridization signal was also observed at P7 but, at this stage, presumed horizontal cells showed a more pronounced appearance. The signal in the entire area of INL also intensified (Figure 4g). A similar pattern was seen at P10 and P15 (Figure 4h,i) with strong cellular labeling, especially at P10. Finally, there was a sharp decline at P21, where only faintly labeled cells were detectable (Figure 4j). To confirm that mir-9 signals belong to specific cell types, co-detection of mir-9 and neuron-specific marker proteins was applied: calbindin for horizontal cells; calretinin for amacrine and ganglion cells (Figure 5).

Horizontal cells were detected at all ages (see representative images for P1 (Figure 5a), P7 (Figure 5b) and P15 (Figure 5c)), and labeling of some amacrine cells was also observed, especially at P3 (Figure 5d) and P7 (Figure 5b,e).

**Figure 6:**
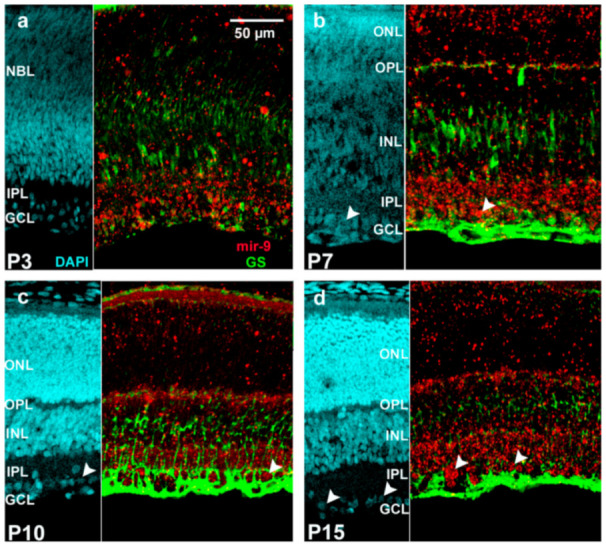
Representative images of miR-9 (red: TSA Cy 3) co-detection with Müller glia-specific marker protein (glutamine synthetase (GS); green: Alexa Fluor-488) at the indicated postnatal days ((**a**) P3, (**b**) P7, (**c**) P10, (**d**) P15). Non-co-localized cells are demonstrated by arrowheads. The scale bar represents 50 µm. GCL, ganglion cell layer; IPL, inner plexiform layer; NBL, neuroblast layer; INL, inner nuclear layer; OPL, outer plexiform layer; ONL, outer nuclear layer.

From the literature, it is known that mir-9 is a Müller cell-specific microRNA [34]. Based on that, we also performed an immunofluorescence staining with the Müller-cell-specific marker glutamine synthetase (GS) in P3 (Figure 6a), P7 (Figure 6b), P10 (Figure 6c) and P15 (Figure 6d) retinas to follow up its expression. GS staining was most intensive in P10 retinas but co-localization with mir-9 could not be confirmed unequivocally.

According to miRTarBase [47] mir-9, among others, potentially targets the transcription factor (OneCut2) [45] and an integral membrane protein of synaptic vesicles (synaptotagmin-17, also known as brain/kidney (B/K) protein) [46]. These abovementioned proteins were documented by immunocytochemistry combined with mir-9 in situ hybridization.

**Figure 7:**
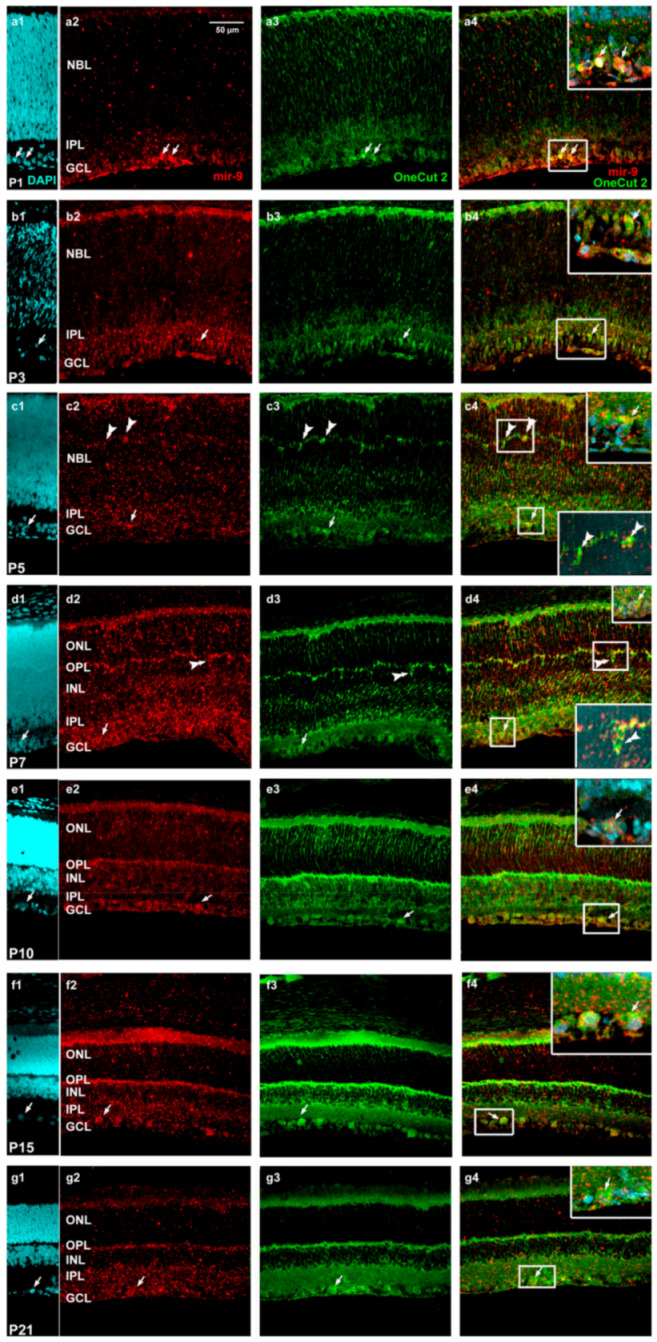
Characterization of miR-9-expressing (red: TSA Cy3) cells (in situ hybridization) and OneCut2 (green: Alexa Fluor-488) by immunolabeling in postnatal developing retina sections at the indicated ages ((**a**1–**a**4) P1; (**b**1–**b**4) P3; (**c**1–**c**4) P5; (**d**1–**d**4 P7; (**e**1–**e**4) P10; (**f**1–**f**4) P15; (**g**1–**g**4) P21). The first column demonstrates the DAPI staining, the second column shows mir-9 and the third column represents OneCut2 staining. The fourth column demonstrates the merged images of ISH–IHC staining. Arrows indicate ganglion cells, and horizontal cells are demonstrated by double arrowheads. Scale bar, 50 µm in Panel a2. NBL, neuroblast layer; GCL, ganglion cell layer; IPL, inner plexiform layer; INL, inner nuclear layer; OPL, outer plexiform layer; ONL, outer nuclear layer.

Immunofluorescence staining indicated that OneCut2 had vigorous expression patterns during the entire retinal development, especially in the ganglion cell layer (GCL) (Figure 7).

Intensive labeling was also observed in the neuroblast layer (NBL) at P1 and P5 (Figure 7(a1–a4), Figure 7(c1–c4)) but not at P3 (Figure 7(b1–b4)). Additionally, at P3, some amacrine cells were double-labeled, while at P5, horizontal cells were detected with both markers. At P5, OneCut2 had already been detected in the GCL (Figure 7(c3)) and after that, at P7 (Figure 7(d1–d4)) and P10 (Figure 7(e1–e4)), gradually increased in all retinal layers. While still high at P15, the expression of OneCut2 decreased mainly in the nuclear layers (outer nuclear layer (ONL) and INL). By co-staining of mir-9 and OneCut2, overlaps were revealed essentially in the GCL during the entire retinal development.

**Figure 8:**
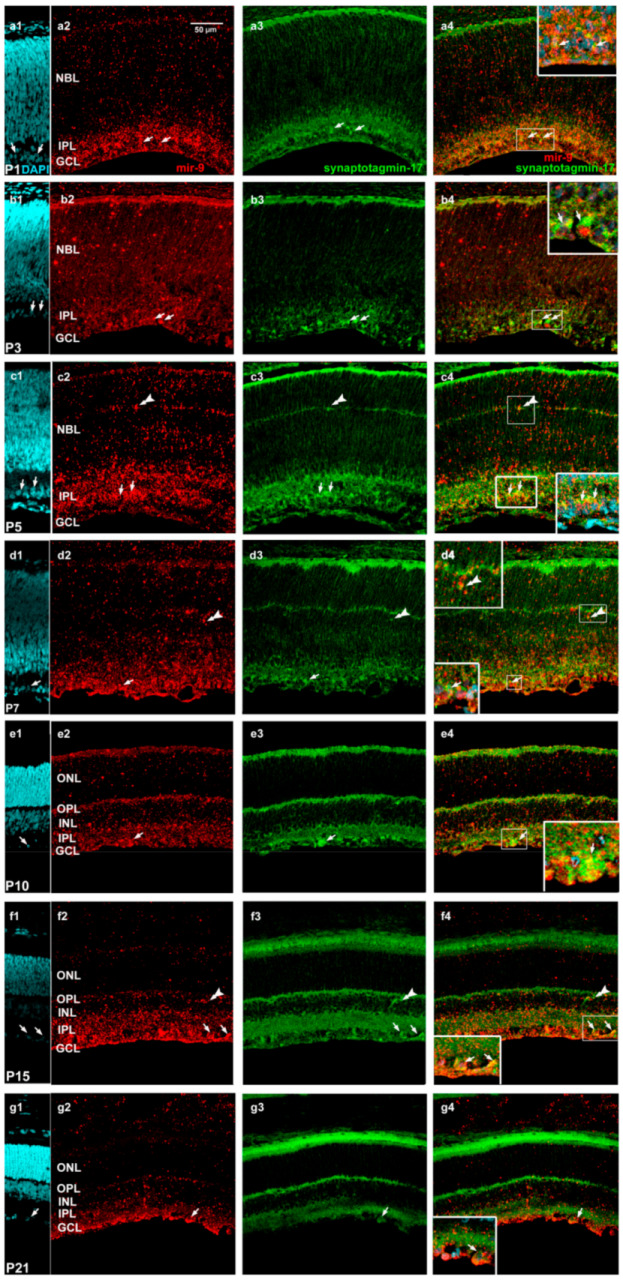
Characterization of miR-9-expressing (red: TSA Cy3) cells (in situ hybridization) and synaptotagmin-17 (green: Alexa Fluor-488) by immunolabeling in postnatal developing retina sections at the indicated ages ((**a**1–**a**4) P1; (**b**1–**b**4) P3; (**c**1–**c**4) P5; (**d**1–**d**4) P7; (**e**1–**e**4) P10; (**f**1–**f**4) P15; (**g**1–**g**4) P21). The first column demonstrates the DAPI staining, the second column shows mir-9 and the third column represents synaptotagmin-17 staining. The fourth column demonstrates the merged images of ISH–IHC staining. Arrows indicate ganglion cells and horizontal cells are demonstrated by double arrowheads. The scale bar seen in Panel a2 represents 50 µm. NBL, neuroblast layer; GCL, ganglion cell layer; IPL, inner plexiform layer; INL, inner nuclear layer; OPL, outer plexiform layer; ONL, outer nuclear layer.

Contrary to OneCut2, synaptotagmin-17 showed only moderate labeling (Figure 8).

At P1 (Figure 8(a1–a4)) and P3 (Figure 8(b1–b4)), synaptotagmin-17 immunoreactivity was characteristic in the inner part of the retina (GCL and the inner part of the INL). At P3, some ganglion cells were double-labeled, while in P5 (Figure 8(c1–c4)) and P7 retinas (Figure 8(d1–d4)), we also detected some horizontal cells with both markers. After P10 (Figure 8(e1–e4)), synaptotagmin-17 intensity gradually decreased in all retinal layers in P15 (Figure 8(f1–f4)) and P21 (Figure 8(g1–g4)) retinas.

## 3. Discussion

It has been previously demonstrated that the retina undergoes several miRNA-dependent changes during development [6,8,10,12,13,14,29]. A large number of existing studies in the broader literature have emphasized that mir-9 is involved and has a crucial role in the control of development, not only in the nervous system [4,25,26,27,28] but also in the retina [9,29,31,48,49]. Xu et al. reported that the expression of mir-9 increased 10-fold from the embryonic state (E10) to adulthood in the mouse retina according to a time-course microarray study. The authors concluded that, among others, mir-9 has a responsibility in the timing of late retinal progenitor cell differentiation into mature retinal neurons and/or Müller glia [29]. During retinal development, overexpression of mir-9 promoted neuron differentiation from RPCs and suppressed glial cell differentiation [35]. In the complex process of retina development, the first two postnatal weeks are critical. The neuronal differentiation begins before birth but the termination of neurogenesis around P5 and the period of synaptogenesis (between P14 and P21) are milestone events [50].

The role of mir-9 in the retinal neurogenesis has been intensively studied in the literature, although little is known about its postnatal distribution and quantity. In our previous in situ methodical study, we saw altered mir-9 expression in the postnatal rat retina [51]. On the basis of these results, in the present work, we try to explain how mir-9 may contribute to postnatal retinal development by applying more methodical approaches and adding more age groups.

Besides mir-9, we chose two other miRNAs (mir-135 for synaptotagmin-17, mir-218 for OneCut2) from our sequencing results, which have also proven to contribute to the regulation of the expression of mir-9′s targets. Contrary to previous studies that demonstrated the gradually increasing expression of mir-9 in the first postnatal week, our qRT–PCR and miRNA-sequencing-based gene expression analyses in the retina showed that this miRNA is upregulated first at P3, but there is a declining tendency to reach a low point at P7; then, at P10, there appears a second peak [29,52]. The other two miRNAs which target the same mRNAs showed a similar tendency to mir-9 expression: their lowest point was at P7; afterwards, they showed an increase. The mir-135 had a peak at P10, while mir-218 had its higher point at P15 and P10. 

The web-based platforms miRTarBase and TargetScan contain broadly annotated, validated miRNA–target interaction datasets. From these we obtained evidence that OneCut2 is a target of mir-9 and mir-218. Mir-218 was also described in the nervous system [53] and shown to regulate the expression of OneCut2 [54]. Several studies have supported the essential role of the members of the OneCut transcription regulator family in development in various parts of the body such as the liver [55,56], the central nervous system [57,58,59] and also in the retina [36,52,60,61]. It has also been revealed that OneCut1 and OneCut2 have similar temporal and spatial expression levels at all stages of retinal development [36]. Although OneCut1 has a higher expression level than OneCut2, both could contribute synergistically to the transition from early to late competence in a dose-dependent manner via regulation of the Lhx1 transcription factor [60]. It is also important to note that earlier investigations tended to assign a role for OneCut 1 and 2 in retinal development but this has not been studied in a comprehensive time-scale resolution postnatally, but only in embryonic phases, emphasizing the effect of OneCut family members on early cell fate regulation [52]. We attempted to explore OneCut2′s appearance by dual qPCR and miRNA-ISH/IHC. Our sequencing results show that mir-9 and mir-218 expression levels had their lowest point at P7; afterwards, they both increased. The OneCut2 expression levels studied by qPCR also decreased until P21. Mir-9 qPCR results also showed higher expression at P3 than in P10, where both the mir-9 and mir-218 sequencing results and the mir-9 qPCR results show peak expression. Our results are in good agreement with earlier examinations showing that OneCut2 is expressed mainly in the GCL. Additionally, horizontal cells in the INL are also able to express this transcription factor postnatally to maintain horizontal cell specification and differentiation [36].

We observed, by in situ hybridization, that mir-9 is highly expressed in the retina, especially in the proximal part. Proposed ganglion and amacrine cells have shown remarkable labeling during the postnatal development during the first 3 weeks; in addition, horizontal cells were also detected. Considering the localization results from in situ hybridization, it seems reasonable to assume that the mir-9 expression detected by qRT–PCR and miRNA sequencing reflects its expression in the RGCs and the cells of the INL of the rat retina.

As has been previously reported in the literature, a single miRNA can regulate an entire set of target genes [2,7]. According to the miRTarBase platform, mir-9 and mir-135 could also target a member of the widely studied synaptotagmin family, namely synaptotagmin-17. Mir-135 expression was also described in the developing and the adult nervous system [53,62,63,64]. Its role in the nervous system was identified as stimulator factor for axon growth and neuron migration. Furthermore, it has a developmentally dependent expression pattern during postnatal development in the mouse brain [62,63]. Both mir-135 and mir-9 showed higher expression levels in Müller cells than in neurons in a microRNA profile study where mir-135 showed increased expression in Müller cells in individuals older than those in the P11 group [34]. In our study, we could not confirm this previous finding, although both mir-9 and mir-135 had a peak at this age. The presence of synaptotagmin-17 in the central nervous system, especially in the brain, has also been widely confirmed [37,38,39,40,41]. These previous investigations revealed that it is an unusual isoform, because of the lack of transmembrane domains, so it is not involved in exocytosis but instead have a crucial role in intracellular membrane trafficking [37,41,42]. However, little research has been conducted to reveal the developmental distribution and expression alterations of synaptotagmin-17 in the rat retina [43,44]. It was observed in the ganglion cells, in a few amacrine cells and in the radial fibers of Müller cells [43]. Although its function is not clear in the retina, it could contribute to B/K protein regulation by the protein kinase A (PKA) intracellular signaling pathway [43,65,66]. Both mir-9 and mir-135 have their lowest point at P7, then increased, where they had the highest expression point at P10. The mir-9 and synaptotagmin-17 qPCR results also showed a decreasing tendency until P7 and, afterwards, they both increased. By combined miRNA-ISH/IHC, we have shown that synaptotagmin-17 is co-expressed with mir-9 postnatally, mostly in ganglion cells and less intensively in amacrine and horizontal cells, and showed the highest expression levels at P3 and P10, while P21 was the lowest point.

Generally, in order to exert its effect, an miRNA must be expressed in the same cells as its mRNA targets [2,7]. The analysis of the cellular co-localization of mir-9 with OneCut2 and synaptotagmin-17 suggests that they are also strongly co-expressed, mainly in ganglion cells and less intensively in amacrine and horizontal cells. Furthermore, our results show that mir-9, mir-218 and mir-135 could act on these targets in a stage-dependent manner. This effect seems to be particularly important on ganglion cells at all stages, on amacrine cells at P3 and P5, and finally on horizontal cells at P5 and P7. Bearing in mind that one mRNA can be targeted by many miRNAs, the translation of a mRNA always depends on the dynamic interaction as well as on the abundance of its miRNAs. It is also well-known that miRNAs can act not only by translational repression but can also have silencing effects or even inducing effects on transcription [67]. According to these facts, our results suggest that mir-9 could induce the transcription of OneCut2 and synaptotagmin-17. However, miRNA–mRNA interaction is only one crucial actor in these molecular processes that affect the expression of these targets. At the same time, they also have a not inconsiderable role in the maintenance and regulation of feedback and feedforward cellular fate, determining molecular networks [67,68].

In conclusion, our results shed some light on the possible stage-dependent transcriptional regulatory role of mir-9 via OneCut2 and synaptotagmin-17 in the postnatal development of the rat retina. However, in order to get deeper insight into the miRNA regulatory process during the postnatal development, additional investigations are necessary (e.g., mir-9 knockout or luciferase assays).

## 4. Materials and Methods

### 4.1. Animal Procedures

Research was conducted in compliance with the ARVO Statement for the Use of Animals in Ophthalmic and Vision Research. Wistar rats aged between postnatal Day 1 (P1) to 21 (P21) were used. Animals were housed under light/dark cycles of 12:12 h with food and water supplied ad libitum. Wistar rats were anesthetized by inhalation using Forane prior to sacrifice. Harvesting processes were executed at the same hour of the day to avoid circadian variations in miRNA expression. Eyes were dissected, and eyecups were prepared and fixed in 4% paraformaldehyde (PFA) for 20 min, cryoprotected, embedded in Shandon Cryomatrix, cut at 12 µm in a cryostat, mounted onto Super Frost Ultra Plus slides and stored at −80 °C until use. In other cases, eyes were removed and then retinas were fixed after dissection in ice-cold RNase-free phosphate-buffered saline (PBS) and stored at −80 °C until use.

### 4.2. RNA Isolation

Small and large RNAs were extracted using a spin column-based method, namely the NucleoSpin miRNA kit (Macherey–Nagel, Düren, Germany) following the manufacturer’s instructions with slight modifications. RNAs were eluted in 50 μL of nuclease-free water pre-heated to 90 °C. An Agilent Bioanalyzer 2100 (Agilent Technologies, Santa Clara, CA, USA) was applied to measure the quality and quantity of the RNA: the Agilent Small RNA Kit for microRNAs and the Agilent RNA 6000 Nano Kit for total RNA. The miRNA content was also measured by the Qubit microRNA Assay Kit (Thermo Fisher Scientific, Waltham, MA, USA).

### 4.3. Small RNA Sequencing and Computational Analyses

Three RNA samples (RNA integrity number (RIN) > 7) from retinas of each age group were pooled and used for sequencing twice according to the manufacturer’s protocols. In brief, pooled RNAs were enriched for small RNAs and then ligated with sequencing adapters. The ligated RNA sample was reverse transcribed, then purified and size-selected with a magnetic bead-based Cleanup Module. Finally, barcodes (Ion Xpress RNA-Seq Barcode 01–16 Kit, Thermo Fisher Scientific, Waltham, MA, USA) were added with the Total RNA-Seq Kit v2 (Thermo Fisher Scientific, Waltham, MA, USA). The quality and quantity of the library were detected via the High Sensitivity Chip of Agilent Bioanalyzer 2100 (Agilent Technologies, Santa Clara, CA, USA). For template preparation, diluted libraries were clonally amplified by emulsion PCR in the Ion OneTouch 2 system (Thermo Fisher Scientific, Waltham, MA, USA) using the Ion PGM Template OT2 200 kit. Templated ISPs were loaded onto an IonTorrent 316 Chip and run on the Ion Personal Genomics Machine (PGM) (Thermo Fisher Scientific, Waltham, MA, USA). Raw files generated by the Ion PGM system (Thermo Fisher Scientific, Waltham, MA, USA) were trimmed, then mapped to the noncoding RNAs from ENSEMBL using the automated pipeline of the Ion Torrent Suite Software. Aligned BAM files were uploaded into a Galaxy web-based platform [69] and the relative abundance of the transcripts was estimated by applying Cufflinks, then several Cufflinks were merged via Cuffmerge. Finally, to detect significant changes in transcript expression, the Cuffdiff application was used [70]. The data have been deposited in the NCBI’s Gene Expression Omnibus [71] and are accessible through GEO Series accession number GSE159168 (https://www.ncbi.nlm.nih.gov/geo/query/acc.cgi?acc=GSE159168, accessed on 1 December 2021). Further analysis and visualization of the datasets were carried out in an R Studio Software environment [72]. The potential target genes of mir-9 were predicted by the miRTarBase online platform (http://mirtarbase.cuhk.edu.cn/php/index.php, accessed on 24 January 2019) [47].

### 4.4. Reverse Transcription and Real-Time PCR Quantitation

For miRNA detection, two-step qPCR was carried out. Briefly, cDNA synthesis from 10 ng RNA using the TaqMan MicroRNA Assay Kit (Thermo Fisher Scientific, Waltham, MA, USA) and qPCR using the StepOne Plus qPCR System (Thermo Fisher Scientific, Waltham, MA, USA) was performed according to the manufacturer’s instructions. The miRNA-specific TaqMan MicroRNA Assay was applied (mir-9: Assay ID 000583) and U6 (Assay ID 001973) was used as an endogenous control. The relative expression levels of each miRNA were determined by using the 2^−ΔΔCt^ method [73] and log2-transformed mir-9/U6 expression ratios were used for further analysis.

For mRNA analysis, 1 μg of total RNA was used with the RevertAid Reverse Transcriptase (Thermo Fisher Scientific, Waltham, MA, USA) and random hexamer primers to generate cDNA according to the manufacturer’s instructions. The predicted targets of mir-9 were amplified using gene-specific TaqMan assays (OneCut2: Rn01265320_m1; synaptotagmin-17: Rn01507712_m1). The thermal profile was as follows: 95 °C for 10 min of initial denaturation followed by 40 cycles of 95 °C for 15 s to denature and 60 °C for 60 s to anneal and extend the product. Relative product quantities were determined using Step One software and the 2^−ΔΔCt^ analysis method [70] using glyceraldehyde 3-phosphate dehydrogenase (GAPDH; Rn01749022_g1) as an endogenous control; RQ values are presented ± SEM (one-way ANOVA following Tukey’s post hoc analysis; * *p* < 0.05, ** *p* < 0.01, *** *p* < 0.001).

### 4.5. Combined MicroRNA In Situ Hybridization and Immunocytochemistry

Slides stored for localization investigation were used to detect mir-9 (Exiqon, Cat#: 88078-01, sequence: TCATACAGCTAGATAACCAAAGA) expression patterns by implementing our previously described in situ hybridization (ISH) method in retinal tissue sections [50]. Briefly, the sections were post-fixed in 4% paraformaldehyde (PFA) for 10 min, treated with 5 mg/mL proteinase K (Sigma-Aldrich, Budapest, Hungary), acetylated in acetic anhydride/triethanolamine and pre-hybridized in a hybridization solution for 4 h (50% formamide, 0.3 M sodium-chloride (pH 7.0), 20 mM Tris-HCl; 5 mM EDTA, 1× Denhardt’s solution, 0.5 mg/mL yeast tRNA, 1× Denhardt’s solution). The tissues were then hybridized with 5′ DIG-labeled LNA detection probe in a hybridization solution at 30 °C below RNA probe Tm overnight. As negative controls, slides incubated without a microRNA probe or without anti-DIG horseradish peroxidase were used. Following hybridization, sections were washed with saline-sodium citrate (SSC) at 65 °C and incubated at room temperature with a blocking solution (30 min, Roche, Welwyn Garden City, UK), followed by a second block in a TNB (Tris–NaCl blocking) buffer (0.1 M Tris-HCl (pH 7.5), 0.15 M NaCl, 0.5% Blocking Reagent (Roche, Budapest, Hungary)).

Sections were incubated with a mouse anti-DIG horseradish peroxidase antibody (1:500, Perkin Elmer, Per-Form Hungary Kft., Budapest, Hungary) and with rabbit anti-calbindin-D28K (1:1000, Swant, Marly, Switzerland; CB38) and rabbit anti-calretinin (1:1000, Swant, Marly, Switzerland; CR7697) or with glutamine synthetase (1:1000, Invitrogen, Thermo Fisher Scientific, Waltham, MA, USA; PA5-28940). For the target immunostaining, we used anti-OneCut2 (1:100, Cambridge, UK; ab28466) or Synaptotagmin-17 (1:100, Abcam, Cambridge, UK; ab220261) and we incubated sections in a humidified chamber overnight.

Following TNT (Tris–NaCl–Tween) buffer washes, the primary antibodies were revealed using Alexa Fluor 488 (1:1000, Thermo Fisher Scientific, Waltham, MA, USA A11034). Slides were then thoroughly washed in the TNT buffer (3 × 5 min). The in situ hybridization signals were detected using the tyramide signal amplification system (TSA) according to the manufacturer’s instructions (1:50, Perkin Elmer, Per-Form Hungary Kft., Budapest, Hungary). Slides were washed (TNT, 3 × 5 min) and mounted in Prolong Gold antifade reagent with DAPI before being analyzed in a confocal microscope (Olympus IX 81 inverse platform—Olympus Fluoview FV-1000 Laser Confocal Scanning Microscope; Olympus, Tokyo, Japan).

## Data Availability

The data presented in this study are available in Supplementary material (Tables S1–S3).

## Supplementary Materials

The following are available online at https://www.mdpi.com/1422-0067/22/5/2577/s1, Table S1: Multiple comparison of miR-9 expression at different time points analyzed by ordinary one-way ANOVA with Tukey’s post-hoc test. Table S2: Multiple comparison of OneCut2 mRNA expression at different time points analyzed by ordinary one-way ANOVA with Tukey’s post-hoc test. Table S3: Multiple comparison of synaptotagmin-17 mRNA expression at different time points analyzed by ordinary one-way ANOVA with Tukey’s post-hoc test.

## Abbreviations

CNS	central nervous system
RPC	retinal progenitor cells
Tbx	T-box transcription factor
Irx	Iroquois homeobox
INL	inner nuclear layer
GCL	ganglion cell layer
IPL	inner plexiform layer
OPL	outer plexiform layer
ONL	outer nuclear layer
NBL	neuroblast layer
RT-qPCR	quantitative reverse transcription PCR
TSA	tyramide-signal amplification
ISH	in situ hybridization
PBS	phosphate-buffered saline
anti-DIG- horseradish peroxidase	anti-digoxigenin-horseradish peroxidase
PFA	paraformaldehyde
TNT	Tris–NaCl–Tween
SSC	saline-sodium citrate
GS	glutamine synthetase
TNB	Tris–NaCl blocking
DAPI	4′,6-diamidino-2-phenylindole
PGM	Ion Personal Genomics Machine
