# Supplementary material for: Analysis of mir-9 Expression Pattern in Rat Retina during Postnatal Development
Source: Int J Mol Sci. 2021 Mar 4;22(5):2577. doi: 10.3390/ijms22052577 (PMC7961372; doi:10.3390/ijms22052577)
Supplement: Supplementary file 1 [file ijms-22-02577-s001.pdf]

International Journal of
Molecular Sciences

MDPI

Article

Analysis of mir-9 expression pattern in rat retina during postnatal development

Etelka Pöstyéni [1], Andrea Kovács-Valasek[1]*, Péter Urbán[2], Lilla Czuni[2], György Sétáló Jr.[3], Csaba Fekete[2] and Róbert Gábriel[1]*

[1] Experimental Zoology and Neurobiology, University of Pécs, 7624 Pécs, Hungary; etelka91@gamma.ttk.pte.hu (E.P.); valasek@gamma.ttk.pte.hu (A.K.-V.), gabriel@ttk.pte.hu (R.G.)
[2] János Szenthágotai Research Centre, Pécs, 7624 Pécs, Hungary; urpe.89@gmail.com (P.U.); czuni.lilla@gmail.com (L.C.), fekete@gamma.ttk.pte (C.F.)
[3] Department of Medical Biology, Medical School, University of Pécs, 7624 Pécs, Hungary; gyorgy.setalo.jr@aok.pte.hu (G.S.)
* Correspondence: gabriel@ttk.pte.hu, valasek@gamma.ttk.pte.hu

International Journal of *Molecular Sciences*

MDPI

Table S1. Multiple comparison of miR-9 expression in different time points analyzed by ordinary one-way ANOVA with Tukey's post-hoc test. Upper part represents data obtained from RT-qPCR, while lower section shows miRNA-sequencing data. (* p<0.05, ** p<0.01, *** p<0.001)

RT-qPCR

	**P1**	**P3**	**P5**	**P7**	**P10**	**P15**	**P21**
**P1**		*** <0.0001	ns	ns	* 0.0274	ns	ns
**P3**			*** <0.0001	*** 0.0003	ns	* 0.0145	*** <0.0001
**P5**				ns	ns	ns	ns
**P7**					ns	ns	ns
**P10**						ns	** 0.0072
**P15**							ns

miRNA-Seq

	**P1**	**P3**	**P5**	**P7**	**P10**	**P15**	**P21**
**P1**		ns	ns	ns	* 0.0143	ns	ns
**P3**			ns	ns	** 0.0061	ns	ns
**P5**				ns	*** 0.0005	ns	ns
**P7**					*** 0.0002	* 0.0219	ns
**P10**						* 0.0194	*** 0.0006

International Journal of *Molecular Sciences*

MDPI

Table S2. Multiple comparison of OneCut2 mRNA expression in different time points analyzed by ordinary one-way ANOVA with Tukey's post-hoc test. (* $p<0.05$, ** $p<0.01$, *** $p<0.001$)

	**P1**	**P3**	**P5**	**P7**	**P10**	**P15**	**P21**
**P1**		ns	ns	* 0.0497	** 0.0041	*** 0.0005	*** <0.0001
**P3**			ns	ns	* 0.0492	** 0.0096	*** <0.0001
**P5**				ns	ns	ns	*** 0.0008
**P7**					ns	ns	* 0.0297
**P10**						ns	ns
**P15**							ns

International Journal of *Molecular Sciences*

Table S3. Multiple comparison of synaptotagmin-17 mRNA expression in different time points analyzed by ordinary one-way ANOVA with Tukey's post-hoc test. (* p<0.05, ** p<0.01, *** p<0.001)

	**P1**	**P3**	**P5**	**P7**	**P10**	**P15**	**P21**
**P1**		ns	ns	ns	ns	ns	* 0.026
**P3**			ns	ns	ns	ns	ns
**P5**				ns	ns	ns	ns
**P7**					ns	ns	ns
**P10**						ns	ns
**P15**							* 0.031
